# Malignant Chondroblastoma of the Os Calcis

**DOI:** 10.1080/13577149878154

**Published:** 1998-03

**Authors:** Emil M. Elek, Robert J. Grimer, David C. Mangham, A. Mark Davies, Simon R. Carter, Roger M. Tillman

**Affiliations:** The Royal Orthopaedic Hospital Oncology Service The Royal Orthopaedic Hospital Birmingham B31 2AP UK

## Abstract

*Patient.* We describe a case of chondroblastoma of the os calcis which metastasized to the tibia, soft tissues and lung. A complete response of the lung lesions was noted with chemotherapy.

*Discussion.* Review of the published literature shows that metastatic chondroblastoma only arises following local recurrence of the tumour.


*Sarcoma* (1998) **2**, 45–48

CASE REPORT

### Malignant chondroblastoma of the os calcis

EMIL M. ELEK, ROBERT J. GRIMER, DAVID C. MANGHAM, A. MARK DAVIES, SIMON R. CARTER & ROGER M. TILLMAN

*The Royal Orthopaedic Hospital Oncology Service, The Royal Orthopaedic Hospital, Birmingham, UK*

**Abstract**

*Patient*. We describe a case of chondroblastoma of the os calcis which metastasized to the tibia, soft tissues and lung. A complete response of the lung lesions was noted with chemotherapy.

*Discussion*. Review of the published literature shows that metastatic chondroblastoma only arises following local recurrence of the tumour.

**Key words**: *chondroblastoma, os calcis, lung metastases.*

### Introduction

Chondroblastomas are recognized as benign primary bony lesions, first described as a distinct entity by Jaffe and Lichtenstein in 1942.[1]

These are rare tumours, the incidence being less than 1% of all primary bone tumours.[2,3] Males are affected more often than females, the ratio being approximately 1.7 : 1.[2] Chondroblastomas are usually located in the epiphysis and are most commonly diagnosed in the second decade, while the epiphyseal plate is still open.[2,4]

The most common sites are the proximal humerus, femur and proximal tibia. They occur less frequently around the ankle or in association with the apophyses.[2–4]

The radiological features are fairly typical: a lytic lesion arising in the epiphysis with an eccentric location, mostly without periosteal reaction. The lesion usually involves less than half of the epiphysis and has a thin sclerotic border.[2,3] Clinically, patients present with pain, swelling around the joint and a limited range of movements due to pain. At a later stage, the tumour could be associated with a synovitis and a joint effusion. Histologically the tumour is built up of round or polygonal chondroblasts surrounded by reticulin fibres. The matrix is pink-stained chondroid, with occasional focal calcification. Scattered osteoclast-type multi-nucleated cells are often present.[2,3]

The usual treatment is simple curettage of the lesion, with occasional bone grafting. Most authors agree that, with intralesional curettage of the tumour, the local recurrence rate is between 5 and 16%.[2,4] Only a few cases have been reported in the literature[5–15] which have metastasized to the lungs, other bones or soft tissues.

In this report, we present the case of a 12-year-old boy with a chondroblastoma of the os calcis. Following surgical treatment the tumour behaved in a malignant manner, with a local recurrence and metastatic disease.

### Case history

In March 1995, a 12-year-old boy was referred to our unit with a 3-month history of pain and swelling of his right ankle region. Radiographs revealed a lytic expansile lesion of the os calcis containing calcification (Fig. 1). Biopsy was carried out prior to referral and histology was consistent with a chondroblastoma. A careful curettage was performed with burring and brushing of the walls of the cavity and pulsed lavage irrigation.

Histology of the curettage specimen showed some necrotic areas and discrete reactive changes due to the previous biopsy, but was fully consistent with chondroblastoma.

Review at 6 weeks was satisfactory with absence of pain or swelling and radiographs showed some early signs of consolidation of the os calcis. Three months later the patient returned with further swelling and pain of the hind foot, with limitation of movements of the ankle and subtalar joints. Radiographs now showed total destruction of the os calcis

Correspondence to: R. J. Grimer, The Royal Orthopaedic Hospital Oncology Service, The Royal Orthopaedic Hospital, Birmingham B31 2AP, UK. Tel: +44 121 685 4150; Fax: +44 121 685 4146.

1357-714X/98/040045-04 ©1998 Carfax Publishing Ltd

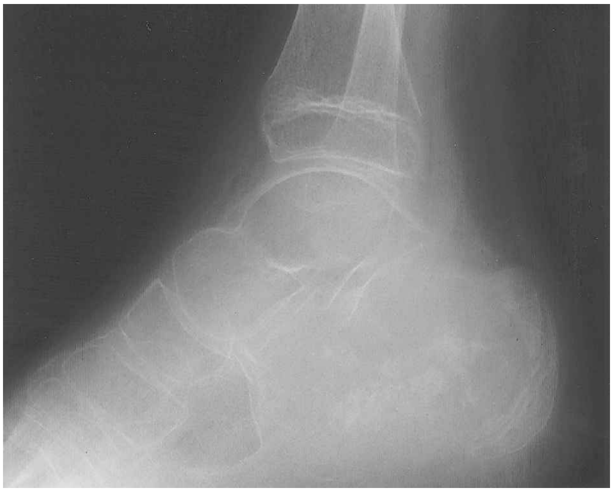

**Fig. 1.** *Lateral radiograph of ankle and hindfoot showing a lytic expansile lesion of the calcaneus containing some cartilage mineralization.*

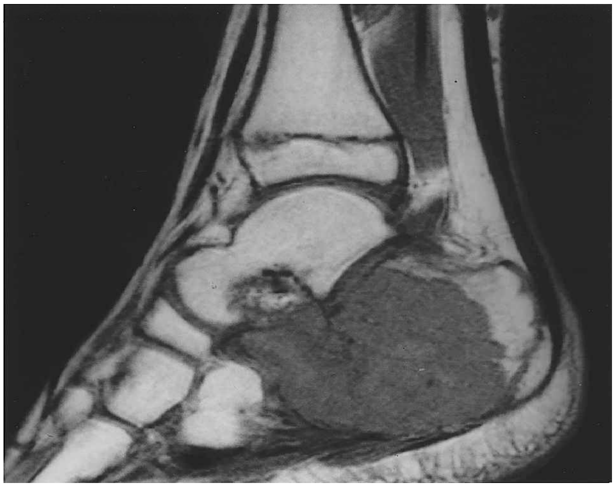

**Fig. 2.** *Sagittal T1-weighted magnetic resonance image of the hindfoot $4\frac{1}{2}$ months after curettage showing extensive infiltration of the calcaneus by recurrent tumour.*

and magnetic resonance imaging (MRI) revealed dramatic extension of the lesion into soft tissues and adjacent bones (Fig. 2). Chest X-ray was normal and a repeat biopsy was performed.

All previous histology slides were reviewed together with the new specimens. Typical chondroblasts were seen in a rich chondroid matrix (Fig. 3). A fairly high mitotic activity (more than 10 mitoses per high-power field) was also seen along with unusual extended necrotic zones. The specimens were reviewed by pathologists in the UK and the US and the consensus was that the recurrent lesion was consistent with a diagnosis of chondroblastoma with atypical features.

As no reconstruction was felt possible the patient was prepared for a below-knee amputation. On admission for this procedure the patient complained of shin pain, and radiographs and MRI now revealed two lytic lesions in his right tibia and a soft tissue mass in the right calf. Biopsy of these lesions was carried out and all the lesions were histologically identical to the appearances of the previous biopsies. Further scans (computed tomography (CT) chest and Tc isotope bone scan) excluded the presence of any other secondary tumours. An above knee amputation was performed.

Histology of the amputation specimen confirmed secondary lesions in the peroneal muscles and several places in the soft tissues of the amputated leg. Histology of each tumour foci was identical to the former biopsies (Fig. 4).

He was restaged 12 weeks later following the amputation when CT scans revealed the presence of lung metastases. In view of this, he was treated with chemotherapy (cisplatin 100 mg $m^{-2}$ and doxorubicin 75 mg $m^{-2}$) and after six cycles there was complete resolution of the lung disease. He remained disease free for over 12 months before relapsing with further pulmonary disease. This was resected surgically and histology again showed identical findings to the previous lesions in the leg. He has had post-operative whole-lung radiotherapy and remains alive and symptom free 3 years from diagnosis.

### Discussion

Since Jaffe and Lichtenstein first described chon-

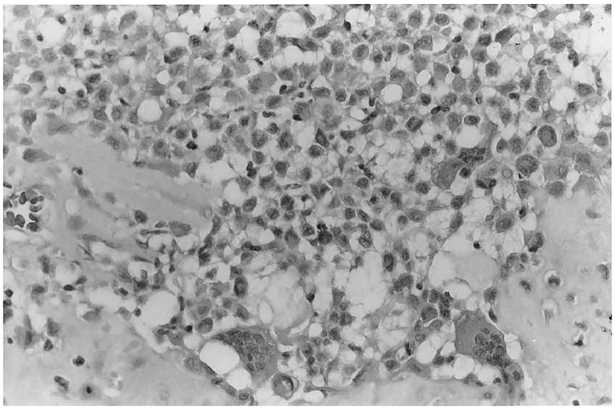

**Fig. 3.** *High power view of primary tumour showing areas of pink chondroid, polygonal 'chondroblast' tumour cells with folded and clefted nuclei and osteoclast-like giant cells.*

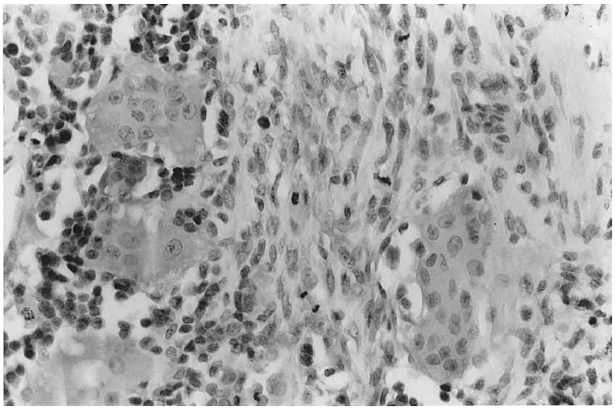

**Fig. 4.** *High power view of recurrent tumour in the calf showing identical features to the original tumour.*

**Table 1.** *Details of reported cases of 'metastatic' chondroblastoma*

| Author | Age | Site | Local recurrence? | Time to metastases | Treatment of metastases | Result |
|---|---|---|---|---|---|---|
| Kahn, 1969[8] | 13 | Pelvis | Yes × 4 | 9 years | Nil | Death 6 years |
| Wellman, 1969[15] | 17 | Scapula | Yes | 25 years | Thoracotomy | Alive 1 year |
| Riddell, 1973[10] | 14 | Tibia | Yes | 2 years | Thoracotomy | Alive 9 years |
| Green, 1975[5] | 13 | Prox. femur | Yes | 2 years | Biopsy only | Alive 2 years |
| Huvos, 1977[7] | 16 | Distal femur | Yes | 1 year | Thoracotomy | Alive 6 years |
| Wirman, 1979[14] | 38 | Scapula | Yes | 33 years | Nil | Death 9 months |
| Sabek, 1982* | 28 | Talus | Yes | 3 years | Not recorded | Not recorded |
| Donovan, 1982* | 15 | Distal femur | Yes | 1 year | Thoracotomy | Alive 5 years |
| Kunze, 1987[13] | 33 | Pelvis | No** | 13 years | Thoracotomy | Alive 6 years |
| van Horn, 1990[12] | 38 | Distal femur | Yes | 11 years | Thoracotomy | Alive 1 year |
| Elek, 1998 | 12 | Os calcis | Yes | 3 months | Chemotherapy | Alive 3 years |

*These patients both quoted in paper of Kyriakos *et al.*[9]
**This patient had a hindquarter amputation following a misdiagnosis of chondrosarcoma but the tumour was subsequently reclassified as chondroblastoma.

droblastoma, several hundred cases have been reported, the vast majority of them confirming that the lesion is clinically benign.

Only a few cases have been reported where, despite the benign histological appearance, the tumour behaved aggressively, occasionally metastasizing to neighbouring or distal bones, soft tissues, or more often the lungs.[5–13] In Sirsat's case[11] the tumour changed to a frankly malignant fibrosarcoma but in all the others the histological appearance of the metastases was identical to the primary tumour.

There appears to be some confusion in the literature about the name malignant chondroblastoma. Some authors consider locally aggressive chondroblastomas to be malignant forms, although there was no evidence of metastasis of the tumour.[1] There is a report of a case where a histologically benign looking chondroblastoma progressively increased in size with secondary spread to the lungs, but following surgery the lung metastases remained unchanged in number and size.[5]

Huvos and Mirra[2,7] suggest that these lung metastases represent self-limited 'transplants' or 'implants', and suggest the term 'benign' metastasis. Kyriakos *et al.*[9] suggest the name 'metastasizing chondroblastoma', rather than malignant chondroblastoma, for these cases.

All the reported cases of confirmed metastatic lesions to the lungs where the histological appearance of the metastases was identical with the primary tumour are identified in Table 1. The *single* common feature of all these cases is that the metastases only developed if there had been a previous local recurrence of the primary tumour. The age and site of the tumours correspond with the normal distribution of chondroblastomas. The only case where there was not a local recurrence was where the primary tumour had clearly been overtreated by a hindquarter amputation after a biopsy had (incorrectly) been interpreted as a chondrosarcoma.[13]

The treatment of the metastases has varied. In some cases the multiplicity of the lesions led to a lack of treatment and was followed by death. In other cases, thoracotomy has produced apparent long-term cures, while in other cases the lesions have remained static following simple biopsy. Clearly, the biological behaviour of these metastatic tumours is unpredictable and is remarkably similar to that seen in metastatic giant cell tumours.[16]

Chondroblastomas represent less than 1% of all bone tumours and the number of patients with documented metastases is less than 1% of all reported cases of chondroblastoma in the world literature. The risk of metastases developing in chondroblastoma is clearly low—and probably much less than 1% of all of these tumours.

From our review of the literature, we conclude that:

(1) Lung metastases have only been reported following local recurrence of the tumour (unless the patient has had an amputation).
(2) Full staging with chest radiographs and/or CT scan of the chest is advisable in any locally recurrent chondroblastoma. Bone scintigraphy and MRI will also be required to exclude spread to other bones or soft tissues, respectively.
(3) In the presence of rapidly progressive chondroblastoma, chemotherapy may be indicated.

### References

1 Jaffe HL, Lichtenstein L. Benign chondroblastoma of bone. A reinterpretation of the so-called calcifying or chondromatous giant cell tumour. *Am J Pathol* 1942; 18:969–91.
2 Mirra JM. *Bone tumours*. Philadelphia: Lea & Febiger, 1989.
3 Vizkelety T, Szendroi M. Csont-Izuleti daganatok es Daganatszeru elvaltozasok. *Medicina Budapest* 1990.
4 Bloem JL, Jacob DM. Chondroblastoma: a clinical and radiological study of 104 cases. *Skeletal Radiol* 1985; 14:19.
5 Green P, Whittaker RP. Benign chondroblastoma.

Case report with pulmonary metastasis. *J Bone Joint Surg (Am)* 1975; 57A:418–20.
6 Hull MT, Gonzalez-Crussi F, DeRosa GP, *et al.* Aggressive chondroblastoma. Report of a case with multiple bone and soft tissue involvement. *Clin Orthop* 1977; 126:261–5.
7 Huvos AG, Higinbotham NL, Marcove RC, *et al.* Aggressive chondroblastoma. Review of the literature on aggressive behaviour and metastases with a report of one case. *Clin Orthop* 1977; 126:266–72.
8 Kahn LB, Wood FM, Ackerman LV. Malignant chondroblastoma. Report of two cases and review of the literature. *Arch Pathol* 1969; 88:371–6.
9 Kyriakos M, Land VJ, Penning LH, *et al.* Metastatic chondroblastoma. Report of a fatal case with a review of the literature on atypical, aggressive, and malignant chondroblastoma. *Cancer* 1985; 55:1770–89.
10 Riddell RJ, Louis CJ, Bromberger NA. Pulmonary metastases from chondroblastoma of the tibia. Report of a case. *J Bone Joint Surg (Br)* 1973; 55-B:848–53.
11 Sirsat MV, Doctor VM. Benign chondroblastoma of bone. Report of a case of malignant transformation. *J Bone Joint Surg (Br)* 1970; 52-B:741–5
12 van Horn JR, Vincent JG, Wiersma-van Tilburg AM, *et al.* Late pulmonary metastases from chondroblastoma of the distal femur. *Acta Orthop Scand* 1990; 61:466–8.
13 Kunze E, Graewe T, Peitsch E. Histology and biology of metastatic chondroblastoma. *Path Res Pract* 1987; 182:113–20.
14 Wirman JA, Crissman JD, Arow BJ. Metastatic chondroblastoma. *Cancer* 1979; 44:87–93.
15 Wellman KF. Chondroblastoma of the scapula. A case report with ultrastructural observations. *Cancer* 1969; 24:408–16.
16 Bertoni F, Present D, Enneking WF. Giant cell tumor of bone with pulmonary metastases. *J Bone Joint Surg (Am)* 1985; 67:890–900.
